# Evaluation of Potential Sustainable Bedding Substrates Focusing on Preference, Behavior, and Stress Physiology in Rats—A Pilot Study

**DOI:** 10.3390/ani11051375

**Published:** 2021-05-12

**Authors:** Miriam Annika Vogt, Lisa Marie Joy Geiger, Talia Härtel, Philipp Follert, Rupert Palme, Sabine Chourbaji

**Affiliations:** 1 Interfaculty Biomedical Research Facility (IBF), University Heidelberg, 69117 Heidelberg, Germany; Geiger@stud.uni-heidelberg.de (L.M.J.G.); Talia.Haertel@gmx.de (T.H.); follert@uni-heidelberg.de (P.F.); chourbaji@uni-heidelberg.de (S.C.); 2 Unit of Physiology, Pathophysiology and Experimental Endocrinology, Department of Biomedical Sciences, University of Veterinary Medicine, 1210 Vienna, Austria; rupert.palme@vetmeduni.ac.at

**Keywords:** bedding, animal welfare, rats, sustainability, stress physiology, corncob, spelt

## Abstract

**Simple Summary:**

The use of animals in research is currently a very controversial and emotionally discussed topic; however, to date, animals still play a pivotal and fundamental role in the advancement of biomedical research. Housing conditions of laboratory animals are especially important, as they constitute an overarching factor independent of the respective experiments. In this pilot study, we investigated different types of bedding, i.e., substrates which are used to house laboratory rats. Bedding is a crucial environmental factor for rats as it has a significant influence on their well-being and the animals tend to have a lifelong exposition to a single type of substrate. Our objective for this pilot study was to evaluate corncob and spelt as ecological alternatives to the most commonly used aspen wood chip bedding. Although the rats’ behavior was not changed by different types of bedding substrates, we detected that the rats liked to consume spelt, which could potentially lead to confounding results, when the animals are tested for certain scientific hypotheses. Therefore, this is a crucial factor to consider when planning an experiment that fulfills high-quality scientific outcomes while simultaneously complying with important animal welfare considerations.

**Abstract:**

Ensuring optimal housing conditions for laboratory animals is a crucial prerequisite for high-quality and ethically justifiable in vivo science. In addition to guaranteeing animal welfare and promoting scientific validity, environmental sustainability is also increasingly gaining attention in laboratory animal facilities. Consequently, comprehensive management of such aspects is one of the core tasks of any research vivarium. Hygienic monitoring and adhering to standardized experimental protocols have been highlighted in the past; nevertheless, various environmental aspects of housing animals still need to be evaluated in greater depth. In this pilot study, we aimed at assessing the suitability of spelt and corncob as economical and ecologically friendly bedding substrates as compared with commonly used aspen wood chips. Therefore, following a descriptive study design, we examined the preferences of male and female Wistar rats for corncob and spelt under specific conditions. In addition, we evaluated potential effects on behavior, metabolism, and stress physiology. The type of bedding did not seem to influence behavior in the observed parameters but did have time- and sex-dependent effects on blood glucose. Furthermore, housing animals on spelt led to a significant reduction in food consumption, probably compensated for by the intake of spelt, and although it did not influence glucose levels, it may have certainly impacted the nutrient supply. Our descriptive pilot study, therefore, highlights the importance of a thorough condition-associated evaluation of even seemingly marginal environmental factors, when balancing potential cost-benefit advances in sustainability and questions of standardization and reproducibility of experimental protocols.

## 1. Introduction

While many husbandry programs of laboratory rodents apply pragmatic and cost-efficient measures, it is recognized that even minor changes in the environment may exert profound effects on phenotype and experimental outcomes [1,2,3]. In this context, the type of bedding substrates might exert effects on research data by gene–environment interactions [4]. In addition to the consideration of potential artifacts and misinterpretation of data, a major concern should be raised concerning animal health and welfare [5]. Therefore, a careful evaluation of bedding substrate is necessary that considers both aspects: (i) animal welfare and (ii) potential influences on scientific data that may be specific to the parameters of interest [6,7].

The market for laboratory animal bedding has always been extensive, comprised of different types of substrates, for example, wood shavings, paper, corncob, and chips of different compositions and ingredient qualities; quality, herein, referring to moisture content as well as microbiological contamination and effects on traumatic injury healing [8,9].

Because of low cost, wood shavings (composed of fine wood particles) are one of the most preferred bedding materials, however, the type of wood needs to fulfill certain requirements regarding toxicity and potential effects on experimental outcomes [10]. Concerning sustainability, transportation distance from the manufacturer to consumer as well as the re-growing potential of used plants should be considered. It must be kept in mind, however, that a continuous availability and standardized quality needs to be guaranteed.

Corncob bedding is an ecologically friendly alternative substrate, derived from granulated cobs and it is a byproduct of (regional) agricultural processing—an essential factor for the consideration as a sustainable solution. Other advantages of corncob bedding are low dust and reduced spread of allergens, as well as low ammonia levels inside the cages [5,11]. However, a study by Ras et al. described this type of substrate as uncomfortable for rats, which limits its use in rodent facilities [12]. Moreover, corncob bedding may evoke changes in sexual behavior and reproduction, and therefore it is recommended that evaluation studies are conducted in any animal facility where this bedding substrate is used [13].

Spelt is a potential substitute bedding substrate that fulfills most of the essential criteria for a suitable litter and is also considered to be especially sustainable with regard to transportation distance, since it is regionally available. Its consistency is softer, which might encourage nestbuilding behavior, and therefore increases the animal’s comfort. In addition, handling and odor after sterilization are good, and the product costs are reasonable. A caution is that, so far, there have been no systematic evaluations of this type of bedding and its effects on routine handling, data reproducibility, and animal well-being.

In this study, we aimed to assess the suitability of spelt as an emerging ecological and economical bedding substrate alternative, for both male and female Wistar rats, as compared with corncob and aspen wood chips as control bedding. Our approach encompassed two steps: (i) preference analysis and (ii) examination of potential environmental effects due to housing on different substrates. In addition, bedding quality was assessed according to humidity, fluid binding, and weight. We hypothesized that exposition to different bedding substrates affects behavior and stress hormone level (analyzed as FCMs). Putatively, such changes correlate with differences in the duration of time spent in the areas containing one of the two substrates offered in the preference cages.

## 2. Materials and Methods

**Figure 1:**
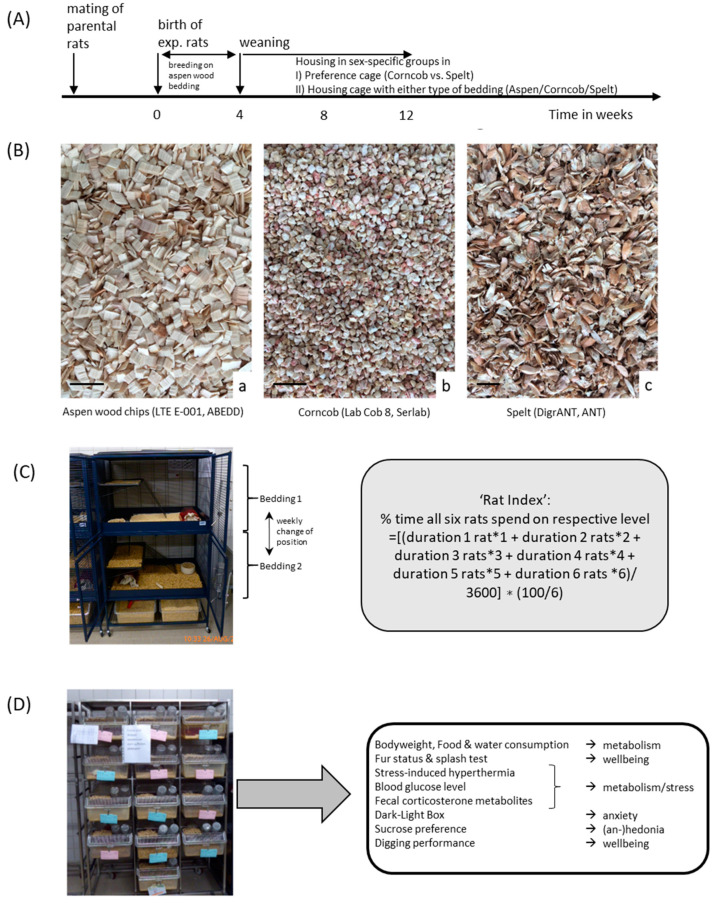
Timeline, bedding material, and experimental set-up. (**A**) For the experiments, after weaning at postnatal Day (PND) 28, the rats were transferred from aspen wood chip bedding to either the preference test setup or type IV cages containing corncob, spelt, or aspen wood chips (control condition); (**B**) illustration of consistency of aspen wood chips, corncob, and spelt bedding substrate, the scale bar represents 1 cm; (**C**) preference test setup to assess ‘rat index’ (RI). Preference cages contained four floors with two different types of bedding, i.e., corncob and spelt, which was changed weekly. The floors were accessible via ladders. Upper and lower cage segments were additionally equipped with red plastic huts and nesting material. Food and water were available ad libitum in upper and lower floors. The ‘rat index’ represents the percentage of time rats spend time on the upper level, and it was calculated by the depicted formula; (**D**) Macrolon^®^ type IV cages were filled with corncob, spelt, and aspen wood chips to analyze the effects on behavior and stress physiology.

Facing the challenge of assessing proper bedding for Wistar rats, we addressed the topic of sustainability by using two wood-free substrates: corncob and spelt (comparison Table 1, Figure 1A,B). All experiments and respective husbandry setups were monitored by local animal welfare officers approved by German competent authorities (Regierungspräsidium Karlsruhe, license number G-182/19).

### 2.1. Animals and Housing Conditions

#### 2.1.1. (A)

In the preference cages, 6 female and 6 male Wistar rats, which were bred and raised in our facility (28 days old at beginning of the experiment) were analyzed. Rats were kept in two four-floor cages in groups of 6 rats separated by sex. The two four-floor cages contained corncob and spelt in the two upper (upper main floor, 75 × 60 cm^2^ and upper mezzanine floor 34 × 60 cm^2^) and lower floors (lower main floor 92 × 60 cm^2^ and lower mezzanine floor 34 × 60 cm^2^), respectively, (cage size 122 × 92 × 60 cm) (Table 1 and Figure 1C). All floors (main and mezzanine floors) could be reached via metal ladders. The environments on the two main floors were slightly different since they were accessibility from different directions. The upper main floor was 17 cm shorter (opening for the ladder from lower mezzanine floor), the lower (but not the upper) mezzanine floor had one ladder in the middle of the floor (accessibility to upper main floor) in addition to the ladder on the side (Figure 1C). The bedding was changed once a week when the cages were cleaned. To avoid preference due to other environmental stimulations, each main floor always included the availability of nesting material, a red plastic tunnel, plus food and water ad libitum (Rod16 feed, Altromin Spezialfutter GmbH & Co. KG, Lage, Germany). The cage lid was covered to prevent the influence of light from above. The night-day cycle was set at 12:12 hours with light on at 07.00 a.m. (approximately 70 lux). Housing and experiments were conducted in a SPF facility at a temperature of 22 °C ± 1 °C and humidity of 55–60%.

#### 2.1.2. (B)

Male and female rats, aged 28 days (directly after weaning), were housed in groups of 3 rats per cage in Macrolon^®^ type IV cages (Figure 1D) (1354G Eurostandard type IV cage, Tecniplast, Buguggiate, Italy) (cage size 598 × 380 × 200 mm and floor size 1820 mm^2^) in the same keeping room as the preference cages. The cages (2 cages per sex and bedding type resulting in *n* = 6 rats per sex and bedding type) contained either (i) corncob (Lab Cob 8, Serlab Ltd. Montataire, France), (ii) spelt (DigrANT, ANT Tierhaltungsbedarf, Buxtehude, Germany) or (iii) aspen wood chips (LTE E-001, ABEDD Vet Lab & Service GmbH, Vienna, Austria) as control substrate on which all rats were raised before weaning (Table 1 and Figure 1B,D). In addition to the weekly assessment of welfare and emotional status during cage changes, the rats underwent a behavioral test battery and stress-induced hyperthermia as well as blood glucose were determined.

### 2.2. Behavioral Analyses

#### 2.2.1. (A) Preference Behavior

Both preference cages with *n* = 6 rats per sex were filmed (ICD-49E CCD Monochrome Camera, Ikegami Electronics (Europe) GmbH, Neuss, Germany) and the preferred substrate of the sex-specific group was determined by a ‘rat index’ (RI). The RI was assessed by relating the number of rats in the cage on respective bedding material provided on the upper cage level, i.e., 100% with all rats (6/6) being present on the preferred bedding substrate, 50% with half of the rats (3/6) preferring one substrate. To avoid artifacts due to preferences for certain cage areas, the bedding was changed every week, i.e., corncob on upper level followed by spelt on the upper level. The RI was calculated by the formula: RI (%) = {[(duration of 1 rat being on the upper level ∗ 1) + (duration of 2 rats being on the upper level ∗ 2) + (duration of 3 rats being on the upper level ∗ 3) + (duration of 4 rats being on the upper level ∗ 4) + (duration of 5 rats being on the upper level ∗ 5) + (duration of 6 rats being on the upper level ∗ 6)/3600] ∗ (100/6)}. The RI analysis was performed twice daily for a duration of one hour (1 h after the start of the light and the dark phase) two times a week in fresh and used bedding (Table 2). The duration of time spent on the upper level was measured manually with the help of the tracking software Ethovision XT (Noldus, Wageningen, the Netherlands), data were always based on the complete measurement time of 1 h. Illumination was 70 ± 10 lux.

#### 2.2.2. (B) Analyses of Metabolism and Behavior Due to Housing on One Type of Bedding Substrate

##### Metabolism: Body Weight Development

All rats were weighed twice per week (*n* = 6 per sex and bedding) and consumption of pre-weighed food (700 g) and water was assessed to measure food and water intake (*n* = 2 measurements per sex and bedding because of cage-wise data).

##### Well-Being: Fur Status and Splash Test

During the weekly cage changing, one rat per cage received a splash (1.2 mL) of 10% dextrose solution on the back fur (*n* = 2 per sex and bedding). Since self-grooming and good fur conditions are an indicator for proper self-hygienic performance, the latency to start self-grooming as well as the duration of stay was measured for 5 min [14].

During cage change, when no splash test took place, we assessed the fur status of each rat (*n* = 6 per sex and bedding) by scoring the state of the fur of 8 distinct body regions: head, neck, dorsal coat, ventral coat, front paws, hind paws, genital region, and tail. A score of ‘0’ was assigned when the status of the body region was well groomed, a score of ‘1’ was set when the fur of a region of the body showed any signs of alteration including fresh bites, wounds, scurf, and fur changes (unkempt, fatty, removed, pinkish, uncleaned). In summary, a minimum score of 0 was assigned for normal and well groomed, and a maximum score of 8 was assigned for a rat with distinct alterations in all 8 distinct body regions.

##### Stress-Induced Hyperthermia

The measurements to assess metabolism (basal body temperature) and hormonal stress response [15] were conducted after Weeks 1, 5, and 8. During manual fixation, body temperature was taken by a digital thermometer, which was rectally inserted (HS digital thermometer VET, Henry Schein Dental, Melville, NY, USA). The procedure was repeated after 45 min and after 2 h to detect potential temperature elevations due to stress. The stressor (additional to the unusual fixation) was the incision of a needle (23G) for blood sampling to analyze blood glucose. This experiment was conducted in one procedure on the same day at around 2.00 p.m. during the light phase for the rats (*n* = 6 per sex and bedding).

##### Blood Glucose Assessment

Assuming that blood glucose levels represent an indicator for stress in many animals [16,17], we performed blood sampling from the tail vessels in parallel with stress-induced hyperthermia, combining the needle incision as a stressor between basal and stress-associated time points. This was done after Weeks 1, 5, and 8 (*n* = 6 per sex and bedding). Blood glucose measurements were performed using a MediTouch monitor (MediTouch, MicrolanceTM3, Medisana, Neuss, Germany).

##### Sucrose Consumption

Anhedonia, a key symptom for depressive-like behavior which can indicate decreased welfare, was assessed during Week 7. For that purpose, the group-housed rats received two bottles in their home cage, one containing regular drinking water and one containing 1% sucrose solution. After 24 h, the weights of both bottles were used to analyze the preference for either type (*n* = 2 per bedding and sex because of cage-wise testing). To avoid side preferences, the positions of bottles were changed after 12 h.

##### Dark-Light Box

The DLB was conducted after Week 8 during the light phase. The rats (*n* = 6 per sex and bedding) were exposed to a box, separated into two compartments for 5 min (each measuring 30 × 20 × 30 cm). In between, the test box was cleaned using 70% alcohol.

We assessed the time needed to enter the brightly illuminated compartment (500 lux) and the length of time the rats spent there. Behavior was monitored by video to avoid direct interference (ICD-49E CCD Monochrome Camera, Ikegami Electronics (Europe) GmbH, Neuss, Germany). To avoid the effects of circadian rhythm, the experiment was conducted over two days during the light phase between 10 a.m. and 1 p.m.

##### Digging Behavior

Digging behavior can be regarded as an indicator of welfare [18,19], and therefore was included in our behavioral test battery. It was conducted during Week 7. Testing involved the placement of conventional conserve glasses (720 mL) into the home cages (*n* = 2 per cage and bedding) which were filled with 300 g food pellets. Latency, frequency, and duration of digging was assessed by analyzing the (videotaped) behavior (ICD-49E CCD Monochrome Camera, Ikegami Electronics (Europe) GmbH, Neuss, Germany).

### 2.3. Feces Collection and Preparation

Levels of fecal corticosterone metabolites (FCMs) were assessed on Day 1 of the experiment (basal level) and after 8 weeks of exposure. To measure stress via a non-invasive technique, cage-wise collection of fecal boli was conducted to avoid isolation stress. All fecal boli inside each group cage (*n* = 2 group cages per sex and bedding) were collected 4 h after cage change and frozen directly at −20 °C. After all experiments were finished, fecal boli were dried at 60 °C for 6 h and homogenized thoroughly by hand. A methanol extraction (80%) was conducted and the extracts stored at −20 °C. FCMs were analyzed with a 5α-pregnane-3β,11β,21-triol-20-one enzyme immunoassay (for details see Touma et al. [20]), which has been proven to be well suited to non-invasively assess adrenocortical activity in rats [21].

### 2.4. Assessment of Bedding Substrate

All bedding substrates were evaluated with regard to weight humidity and possible consumed loss. To assess these parameters, type IV cages were filled with either 650 g aspen wood chips, 1200 g corncob, or 730 g spelt. After 3 days of usage by the 3 groups of rats, the weights of bedding substrate were assessed. Humidity was measured by an electronic analysis instrument (GMH 3850, Greisinger electronic, Remscheid, Germany).

### 2.5. Statistical Analysis

Data from this pilot study were analyzed statistically using Excel and SPSS 26 (IBM). Descriptive data comprise means and standard error of the mean (SEM). Bi-factorial analyses of variance (ANOVA) were conducted comprising the factors ‘bedding’, ‘time’, and ‘sex’ with Tukey’s post hoc analyses. Differences, which were assessed throughout the experimental course, considered repetition of measurements. Potential differences among the groups were examined by means of Student’s *t*-tests. All significant findings were related to a significance level of *p* < 0.05.

Individual rats in experiment B were considered as ‘experimental units’, since the aim of the study was to confirm changes in the level of single data and not cage means for animals with either type of bedding substrate. The bedding could affect the animals at different sex/hierarchy types/emotional states differently, therefore, an individual examination was chosen over a general cage-wise observation. For sucrose consumption, digging behavior, and glucocorticoid levels measured as FCM, the experimental design, however, limited the sample size to *n* = 2 as data of cages were assessed.

## 3. Results

### 3.1. (A) Preference Behavior

**Figure 2:**
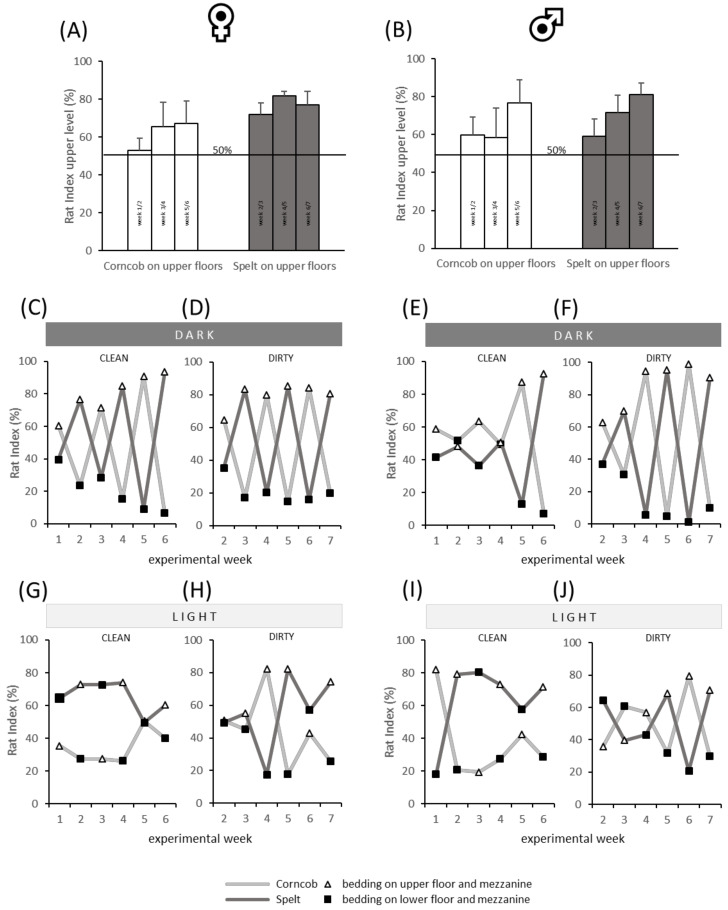
Spatial pattern in the preference cages in the context of different types of beddings and light conditions for female (left) and male (right) rats, illustrated by the rat index (RI). (**A**,**B**) Mean RI (+SEM) for the upper level only, summarizing per column the four different conditions (light/clean, light/dirty, dark/clean, and dark/dirty). Experimental weeks are referring to those in Table 2; (**C**–**F**) RI in dark conditions under clean and dirty conditions, respectively, for females (**C**,**D**) and males (**E**,**F**). Independent of bedding, rats demonstrate descriptively higher preferences for the upper compartment; (**G**–**J**) RI in light conditions under clean and dirty conditions, respectively, for females (**G**,**H**) and males (**I**,**J**). Rats demonstrate descriptively higher preferences for the upper compartment. When looking at clean versus dirty conditions, the illustration indicates a preference for clean spelt under light conditions. Light grey line, corncob; dark grey line, spelt bedding substrate; white triangle, measurement of the upper floor and mezzanine; black square, measurement of the lower floor and mezzanine; each dot represents 1 h measurement.

The analysis of the RI revealed a preference (RI > 50%) for the upper floors in males and females (Figure 2A,B) irrespective of the bedding substrate, light, and clean/dirty condition. Details of the spatial patterns were examined in the context of different conditions, as shown in Figure 2C–J. The preference for the upper floors is particularly obvious in the dark condition, as illustrated by white triangles in Figure 2C–F. Especially in the females (Figure 2C,D) and in the dirty condition of the males (Figure 2F,J), the bedding substrate seems not to play an important role as preference for the upper level increased over time. In light condition, the patterns seem to be more divergent. Especially the clean spelt bedding (dark grey line) is preferred over corncob, independently of the position in the cage in female (Figure 2G) and male rats (Figure 2I). No preference is visually apparent when the bedding substrate had been used for 6 days (dirty condition, Figure 2H,J).

### 3.2. (B) Analyses of Metabolism and Behavior Due to Housing on One Type of Bedding Substrate

#### 3.2.1. Metabolism: Body Weight Development and Food Consumption

All rats, males and females, gained weight throughout the experimental observations (factor time F(15,450) = 2298.2, *p* < 0.001). There was a significant effect of sex (factor sex F(1,30) = 193.54, *p* < 0.001) with females’ weights increasing significantly less than males’ weights (interaction time ∗ sex F(14,450) = 195.1, *p* < 0.001). There was, however, no influence of different bedding substrates on weight development (F(2,30) = 0.13, *p* = 0.881).

**Figure 3:**
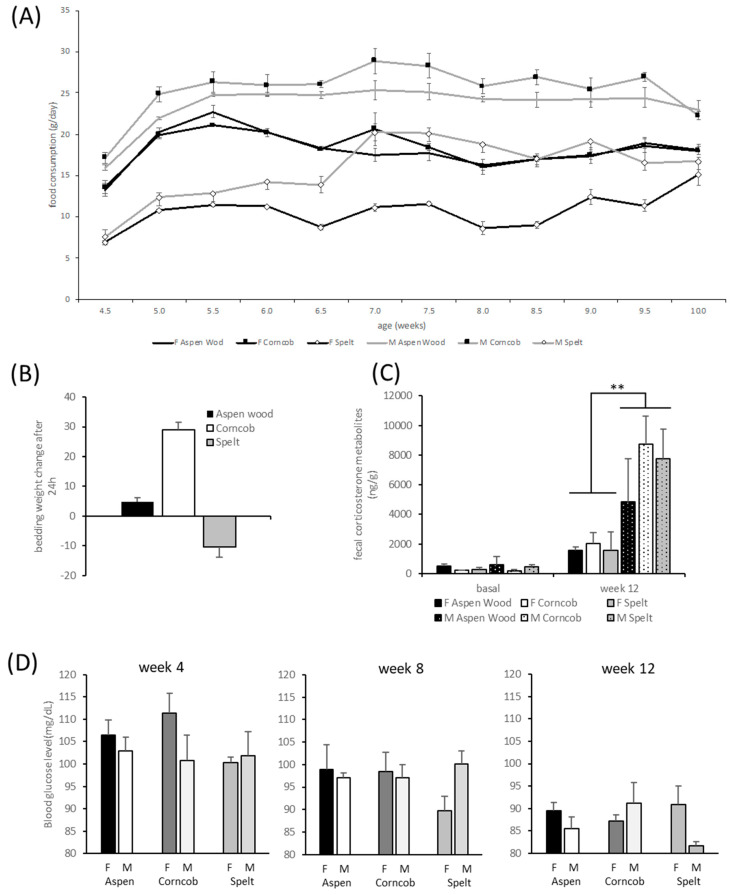
Food consumption, bedding usage, fecal corticosterone metabolites, and blood glucose levels of rats kept on one type of bedding. (**A**) Consumption of food pellets over a period of 8 weeks in males and females varies with the two lower curves representing feeding behavior in males and females housed on spelt bedding; (**B**) aspen wood chip and corncob bedding weight increased after usage, while spelt weight/quantity is reduced after housing rats for 24 h (**); (**C**) Fecal corticosterone metabolites do not differ significantly concerning bedding substrate but are dependent on sex, although this effect is not present at an age of 4 weeks (basally) but is present at an age of 12 weeks; (**D**) Blood glucose levels in males and females, as assessed during Weeks 4, 8, and 12, are reduced over time with varying directions depending on ‘sex’, ‘time’, and ‘bedding’.

Rats consumed more food over time (factor time F(11,66) = 26.75, *p* < 0.001). Female rats ate generally less than males (factor sex F(1,6) = 175.60, *p* = 0.001, Figure 3A). The rats housed on spelt ate throughout all measurements significantly less than the other rats on either aspen wood chips or corncob bedding (interaction time ∗ bedding F(22,66) = 3.59, *p* < 0.001). The factor ‘bedding’ had a significant effect on food consumption (factor bedding F(2,6) = 149.37, *p* < 0.001); the rats housed on spelt consumed significantly less as compared with corncob (*p* < 0.001) and aspen wood chip bedding (*p* < 0.001), as revealed by Tukey’s post hoc testing.

#### 3.2.2. Well-Being/Splash Test

Splash tests as well as thorough monitoring of fur status did not reveal any statistical differences, for males or for females over the time of observations.

#### 3.2.3. Stress-Induced Hyperthermia

Stress-induced hyperthermia varied over time (factor time, F(2,60) = 48.10, *p* < 0.001) and sex (factor sex, F(1,30) = 20.61, *p* < 0.001), indeed there was an interaction of ‘time’ ∗ ‘sex’ (F(2,20) = 10.32, *p* < 0.001).

#### 3.2.4. Blood Glucose Assessment

Throughout the three time points in Weeks 1, 5, and 8, there was an effect of the factor ‘time’ with decreasing blood glucose levels in both sexes, i.e., F(2,4) = 26.83, *p* < 0.001. Depending on the time of measurement, there was an interaction of ‘time’ ∗ ‘bedding’ ∗ ‘sex’ with males and females presenting different quantities of blood glucose (F(4,54) = 2.89, *p* = 0.031, Figure 3D).

#### 3.2.5. Sucrose Consumption

There were no detectable or statistically significant differences related to sex or type of bedding substrate.

#### 3.2.6. Dark-Light Box

There was no influence of the factor ‘bedding’ on anxiety behavior when latency to enter and time spent in light compartment were addressed. However, females demonstrated more entries into the light area (factor sex, F(1,29) = 10.52, *p* < 0.003).

#### 3.2.7. Digging Behavior

The animals did not show any motivation to dig food pellets during the first 15 min of testing. Therefore, no differences in digging behavior could be detected.

#### 3.2.8. Fecal Corticosterone Metabolites (FCM)

Cage-wise collection to avoid isolation stress limited the number of experimental units to *n* = 2 per sex and cage. Basal levels of FCM were not significantly different concerning effects of sex and bedding. After 8 weeks of housing under respective conditions, corticosterone metabolite content in the feces was significantly dependent on sex (F(1,6) = 14.622, *p* = 0.009) with males having higher FCM levels than females, but no significant effect of bedding was detectable on FCM levels (Figure 3C).

#### 3.2.9. Assessment of Bedding Substrate

The decrease in spelt bedding weight was significant for both sexes (Figure 3B, factor sex, F(2,6) = 37.95, *p* < 0.001). A general observation revealed an enhanced increase in bedding weight of males as compared with that of females (factor sex, F(1,6) = 10.14, *p* < 0.019).

## 4. Discussion

It has been established that bedding represents a crucial factor that can have an influence on the well-being of mice, and can also affect the experimental outcome in mice [22]. The bedding substrate primarily serves to standardize the micro-environment of the animals as well as the working conditions for caretakers. Moreover, bedding may be integrated as an essential component in nest building behavior, which is part of the behavioral repertoire of many rodents [9,23,24]. Most of the findings relate to studies in mice, but similar influences may be assumed for rats.

As described in recent studies, certain experimental outcomes may be affected by different types of bedding, for example, slow-wave sleep [25], well-being [26], or ultrasonic vocalization [27]. Our rat study on preferences for and effects of different bedding types on behavior and physiological factors highlights that proper evaluation of environmental factors is an essential aspect to consider when conducting in vivo studies.

As we did not detect a general influence of the tested bedding substrates in our specific behavioral test battery, theoretically using any of the tested bedding types seems to be safe and free of inducing artifacts. However, our study results indicated a sex-specific preference for spelt versus corncob bedding.

By exposing male and female Wistar rats to two commercially available, sustainable, and eco-friendly bedding substrates, we aimed at expanding the current knowledge regarding its effects on the levels described above. Preference tests are commonly used to detect preferences of rodents for certain types of environmental stimuli [28]. Many setups make use of especially designed tunnel cage systems and assess latency and duration of time spent in the experimental environment [28,29]. Our preference test differed from most of the systems in the literature in terms of being a multi-floor system with weekly altered bedding conditions in the upper and lower levels. The purpose of our bedding alteration regime was to avoid potential bias related to elevated position, for example, rats might prefer the upper cage compartment in such settings as described before [30].

Indeed, we reproduced this preference for elevated positions in both males and females, which had previously been interpreted as curiosity behavior. Moreover, rats could favor elevated positions because of hierarchic motivations (not being attacked from above) or to simply have the ideal overview position. Such results suggest that giving animals the chance to actively choose their favorite position is an essential aspect to enrich environmental conditions, which supports species-appropriate behaviors.

However, the focus of the study was to assess the suitability of corncob and spelt as alternative substrate to aspen wood chips. Both sexes displayed average and comparable growth on all substrates. Males exhibited higher body weights than females, an expected finding which has been previously described [31]. Food intake increased over time relative to age and physical development. However, food was partly substituted by spelt intake, which was seen in both sexes. This was a disturbing observation, because metabolic effects of a non-standardized diet cannot be excluded, an issue that has also been described by Clarke et al. in a different setting [32].

No significant bedding substrate-related differences occurred with regard to stress-induced hyperthermia, blood glucose, self-maintenance, or anxiety behavior, and therefore animal well-being seemed not to be affected. Due to the inherent limitation of choosing the method of group-housed rats and the limited scope of this study, an in-depth evaluation of metabolic parameters was not possible. For further evaluation, especially of metabolic or stress physiological data, more animals and experimental units would be necessary.

Interestingly, we did not detect an effect of corncob on anxiety behavior in the DLB, as described by Sakhai et al. [3]. This may be caused by the different time frames during development when rats were exposed to this substrate. Accordingly, our chosen time frame would support the use of this substrate without the risk of inducing artifacts.

## 5. Conclusions

In summary, the results of our study highlight the importance of an explicit awareness when environmental factors in animal husbandry are changed, for example, switching to a more eco-friendly or economical substrate. Since rodents are usually continuously housed on one substrate throughout their lifetime, the choice of bedding substrate needs to be addressed properly and potential side effects need to be ruled out. On the one hand, consumption of spelt bedding substrate, which was observed in our study, can negatively affect well-being over time, since the animals obviously used it as a food alternative that lacks the necessary supplements Additionally, it may state a KO criterium, because of the induced lack of standardization. On the other hand, spelt as an enrichment, may promote a potential benefit because of rats’ persistence to search and dig in the bedding and the subsequent oral manipulation. Corncob and aspen wood chips also present a certain risk of producing artifacts (as every environmental factor does), which needs to be adequately balanced. Certainly, there is no general panacea, and therefore decisions with regard to the environment of rodents have to be made individually, addressing the multifactorial conditions of an animal facility. An interesting question for future studies might be the potential of spelt as an alternative bedding substrate to corncob and aspen wood chips for other rodents, for example, mice.

## Figures and Tables

**Table 1:** Comparative evaluation of the three bedding substrates used in this study. The different aspects are provided by the respective manufacturer.

	Aspen Wood Chips	Corncob	Spelt
Product name	LTE E-001	Lab Cob 8	Digr-ANT
Manufacturer	ABEDD Vet Lab & Service GmbH, Vienna, Austria	Serlab Ltd., Montataire, France	ANT Tierhaltungsbedarf, Buxtehude, Germany
Production details	Barked aspen wood (*Populus tremula*), chipped, dried (110 °C), sifted	Whole corncobs (*Zea mays*) ground, dried, sifted	Husked spelt (*Triticum aestivum* subsp. *Spelta*), pelleted with steam
Region of origin	Riga, Latvia Styria, Austria	Missing information	East- and southern Germany
Size	2–5 mm	95% in range 2–3.15 mm	2–10 mm, max. 12 mm, 80% < 10 mm (estim., dependent on natural occurrence)
Humidity	10–12%	<12%	<8%
Absorption	172–184%	105%	400%
Apparent weight	190 g/L	350 g/L	300 g/L pelleted, 120 g/L (after autoclaving)
Dust content	0.013%	Missing information	<0.1%
Autoclavable	Yes	Yes	Yes (doubling of volume)
Properties	Dust free, odor absorbent	Reducing ammonia generation	Tasteless, odorless

**Table 2:** Time points for the assessment of the preference behavior (‘rat index’) in the preference cages. The analyses of preference behavior started with weaning, when the rats were placed in the new fresh preference cage. The rats were videotaped four times a week, except for Week 1 (no ‘dirty’ condition) and Week 6 (no ‘clean’ condition). *n* = 1 cage per sex (with 6 rats in each cage).

Week	Age of Rats	Day in Week	Time	Bedding Type Upper Level	Status
1	Day 28	day of weaning, fresh cage	7 a.m.–8 a.m.	corncob	clean/light
			7 p.m.–8 p.m.	corncob	clean/dark
2	Day 34	1 day before cage cleaning	7 a.m.–8 a.m.	corncob	dirty/light
			7 p.m.–8 p.m.	corncob	dirty/dark
	Day 35	at day of cage cleaning	7 a.m.–8 a.m.	spelt	clean/light
			7 p.m.–8 p.m.	spelt	clean/dark
3	Day 41	1 day before cage cleaning	7 a.m.–8 a.m.	spelt	dirty/light
			7 p.m.–8 p.m.	spelt	dirty/dark
	Day 42	at day of cage cleaning	7 a.m.–8 a.m.	corncob	clean/light
			7 p.m.–8 p.m.	corncob	clean/dark
4	Day 48	1 day before cage cleaning	7 a.m.–8 a.m.	corncob	dirty/light
			7 p.m.–8 p.m.	corncob	dirty/dark
	Day 49	at day of cage cleaning	7 a.m.–8 a.m.	spelt	clean/light
			7 p.m.–8 p.m.	spelt	clean/dark
5	Day 55	1 day before cage cleaning	7 a.m.–8 a.m.	spelt	dirty/light
			7 p.m.–8 p.m.	spelt	dirty/dark
	Day 56	at day of cage cleaning	7 a.m.–8 a.m.	corncob	clean/light
			7 p.m.–8 p.m.	corncob	clean/dark
6	Day 62	1 day before cage cleaning	7 a.m.–8 a.m.	corncob	dirty/light
			7 p.m.–8 p.m.	corncob	dirty/dark
	Day 63	at day of cage cleaning	7 a.m.–8 a.m.	spelt	clean/light
			7 p.m.–8 p.m.	spelt	clean/dark

## Data Availability

Data is available on request.
